# Nutritional quality of foods and non-alcoholic beverages advertised on Mexican television according to three nutrient profile models

**DOI:** 10.1186/s12889-016-3298-0

**Published:** 2016-08-05

**Authors:** Sofía Rincón-Gallardo Patiño, Lizbeth Tolentino-Mayo, Eric Alejandro Flores Monterrubio, Jennifer L Harris, Stefanie Vandevijvere, Juan A Rivera, Simón Barquera

**Affiliations:** 1 Centro de Investigación en Nutrición y Salud, Instituto Nacional de Salud Pública, Av. Universidad No. 655, CP 62100 Cuernavaca, Morelos México; 2 Rudd Center for Food Policy & Obesity, One Constitution Plaza, Kinsley St, Hartford, CT 06103 USA; 3 School of Population Health, The University of Auckland, Private Bag 92019, Auckland, 1142 New Zealand

**Keywords:** Television, Advertising, Food and beverages, Nutritional content, Mexico, Nutrient profile models

## Abstract

**Background:**

Evidence supports that television food advertisements influence children’s food preferences and their consumption. However, few studies have examined the extent and nature of food marketing to children in low and middle income countries. This study aims to assess the nutritional quality of foods and beverages advertised on Mexican TV, applying the Mexican, World Health Organization (WHO) European and United Kingdom (UKNPM) nutrient profile models, before the Mexican regulation on food marketing came into effect.

**Methods:**

We recorded 600 h on the four national public and free TV channels with the highest national ratings, from December 2012 to April 2013. Recordings were done for 40 randomly selected (week, weekend, school and vacation) days, from 7 am to 10 pm. Nutritional information per 100 g/ml of product was obtained from the product labels or company websites.

**Results:**

A total of 2,544 food and non-alcoholic beverage advertisements were broadcast, for 275 different products. On average, the foods advertised during cartoon programming had the highest energy (367 kcal) and sugar (30.0 g) content, while foods advertised during sport programming had the highest amount of total fat (9.5 g) and sodium (412 mg) content. More than 60 % of the foods advertised did not meet any nutritional quality standards. 64.3 % of the products did not comply with the Mexican nutritional standards, as compared with 83.1 % and 78.7 % with WHO Europe and UKNPM standards, respectively. The food groups most frequently advertised were beverages (24.6 %), followed by chocolate and confectionery sugar (19.7 %), cakes, sweet biscuits and pastries (12.0 %), savory snacks (9.3 %), breakfast cereals (7.1 %), ready-made food (6.4 %) and dairy products (6.0 %).

**Conclusion:**

The majority of foods and beverages advertised on Mexican TV do not comply with any nutritional quality standards, and thus should not be marketed to children. The nutritional quality standards applied by the Mexican regulation are much weaker than those applied by the WHO Europe and United Kingdom. The Mexican government should improve the nutrition standards in its new regulation, especially the sugar cut off points.

**Electronic supplementary material:**

The online version of this article (doi:10.1186/s12889-016-3298-0) contains supplementary material, which is available to authorized users.

## Background

Over the past decades the prevalence of childhood obesity has risen internationally [1] and has been recognized as a global public health problem [2]. Child obesity rates have increased in Mexico as well. The National Health and Nutrition Survey 2012 (ENSANUT, for its acronym in spanish) [3] found that the prevalence of overweight and obesity among children under 5 years increased from 26.6 % to 33.6 % between 1988 and 2012 (0.3 percentage point per year), and from 28.2 % to 36.9 % for children 5-11 years between 1999 and 2012 (0.7 percentage point per year). Childhood obesity is linked to cardiovascular risk factors, type 2 diabetes, dental caries and chronic diseases later in life [4, 5]. This problem has been attributed to numerous causes, including a sedentary lifestyle, an unhealthy diet, socioeconomic status, the physical environment, and integrated food marketing through multimedia [6, 7].

Evidence supports that television (TV) viewing is associated with obesity among children, because it is a major sedentary activity, and also due to children’s responses to the food and beverage advertisements shown on television [8–11]. TV food advertisements influence children’s food preferences and purchase requests, and consequently their consumption and body weight outcomes [5, 12–14]. In addition, foods marketed to children are often high in saturated fat, sugar and/or sodium (HFSS) [11, 15]. Links have been established between the marketing of food products with poor nutritional quality and overweight and obesity among children [16–19].

In 2006, the Institute of Medicine Report on Food Marketing to Children and Youth highlighted the need to address the nutritional content of food products advertised to children [5]. Later, a set of seven principles (the Sydney Principles) were developed by World Obesity (formerly the International Obesity Taskforce (IOTF)) in order to guide action and reduce the promotion of unhealthy foods and beverages to children [20]. In 2010 and 2011, both the World Health Organization (WHO) and the Pan American Health Organization (PAHO) supported policies to regulate child-directed advertising of foods with poor nutritional quality, in an effort to protect children from being exposed to these potentially harmful food advertisements [21, 22]. In July 2013 the Ministers of health and representatives of the Member States of the WHO, signed the Vienna Declaration, which included a commitment to take decisive action to reduce the exposure of children to unhealthy food marketing [23]. In May 2013 all Member States adopted the WHO non-communicable disease (NCD) action plan and monitoring framework, which included the reduction of junk food marketing to children as one of the indicators.

Following the recommendations from these international organizations, Mexico introduced a set of regulations in 2015 to limit unhealthy food and beverage advertising to children on screens, both on TV and in movie theaters [24], as part of the 2013 National Strategy for the Prevention and Control of Overweight, Obesity and Diabetes [25]. The nutritional criteria (Additional file 1) for this new policy were created using the European Pledge [26] as a reference. This pledge was developed by the food industry as a commitment to advertise healthier food choices in child-directed media and promote self-regulation instead of legislation [27]. It is designed as a self-regulatory and voluntary initiative where food products advertised have to contain food category-specific components per definition (e.g. dairy products needs to contain at least 50 % dairy), otherwise they should not be advertised. Additionally, depending on the food category, maximum amounts are set for energy, sodium, saturated fats and total sugars. The Mexican regulation allows advertising of food products that do not comply with the nutritional criteria during specific hours, from Monday to Friday between 12:00 am to 2:30 pm and 7:30 pm and 12:00 am, and Saturday and Sunday between 12:00 am and to 7:00 am and 7:30 pm to 12:00 am. In addition, the nutritional criteria do not apply to those programs targeted to an audience 12 years and older, such as soap operas, sports programing, news, and TV series [24].

Internationally, other nutritional profile models have been developed independently from the food industry to restrict unhealthy food marketing to children, such as a model proposed by the WHO for European countries [28] and one currently used in the United Kingdom [29].

The European nutrient profile model (see Additional file 2) was developed by the WHO Regional Office for Europe, with the objective to use it as a common model to improve the nutritional quality of the foods marketed to children in Members States across Europe [28]. On the other hand, the United Kingdom Profile Model (UKNPM) was developed by the British Heart Foundation Health Promotion Research Group at Oxford University and is currently used in legislation that restricts unhealthy food marketing to children on TV. This model generates a score, which determines whether the food can be advertised to children or not. Two threshold levels are set: one for food products and one for beverages [29].

Assessment of the extent and nature of food marketing to children on TV has previously been performed in Australia, New Zealand, Asia and other countries in Europe, North and South America, but little research has been undertaken in Mexico [15, 30–34]. The current study aims to assess the nutritional quality of food and beverages advertised on Mexican TV, applying the Mexican, WHO Europe and UKNPM standards. The present study was conducted before the Mexican regulation came into effect. The hypothesis of the study is that the majority of the foods and beverages advertised on Mexican television promote products high in fat, sugar and sodium.

## Methods

### Study design

We recorded 600 h of Mexican TV on the four free and public channels with the highest national audience ratings from December 2012 to April 2013 (XHTV Channel 2, XHGC Channel 5, XHIMT Channel 7 and XHDF Channel 13). These channels cover more than 90 % of the Mexican territory [35]. Days were randomly selected over the 5 months, 10 days were recorded per channel from 7 am to 10 pm. These recordings included broadcasts to a wide range of audiences and many different types of programs.

As TV programming is different during school and vacation season periods, we included recordings of advertisements for both of those periods in the same year. The official calendar of the Ministry of Public Education of Mexico was used to classify days into school or vacation season [36], and the target audience of the programme was determined according to the Mexican Federal law of Radio and TV: general audience (A classification) or audience of 12 years and older (classification B, B15, C and D) [37]. The types of programs were categorized as sports, soap operas, cartoons, entertainment, movies, or others (i.e.: news, series, contests, musicals, reality shows and health related programs). The categorization was done using a manual used by the Brazilian Institute of Public Opinion and Statistics (IBOPE, for its acronym in spanish) to measure TV audience [38].

The recordings were captured using 4 digital TV adaptors (WIN TV-HVR / Hybrid Stick 950Q). For coding of the recordings, a manual developed by the Rudd Center for Food Policy & Obesity was used [18]. Personnel were trained to code the advertisements in a standardized manner. Three research assistants participated in the coding.

### Nutritional analysis

The nutritional content of food and beverage products was obtained from the nutrient declarations of each product; the information was captured by photo in 2015. When that was not available, nutritional information was obtained from company websites updated in 2015. Only one product (paleta teleton) was excluded from the nutritional content analysis because its composition could not be obtained from either of these sources. Nutritional assessments were based on energy (kilocalories), sugar, fiber, total fat, saturated fat (all in grams), and sodium (milligrams). Data was collected per 100 g/ml from all the products advertised.

The nutritional quality was assessed according to 3 different nutrient profile models (see Table 1), each designed to assess the healthiness of food and beverage products advertised on TV in an effort to reduce unhealthy food-marketing targeted to children. The three standards were: (i) The nutritional criteria proposed by the Mexican Ministry of Health for the new regulation (Mexican) [24]; (ii) WHO Regional Office for Europe nutrient profile model (European) [28] and (iii) United Kingdom Nutrient Profiling Model (UKNPM) [29]. All products in each advertisement were assessed separately; as*“less healthy or healthier products”* for the three different criteria. We tagged a product as *“less healthy”* when it did not comply with the specific nutrient criteria, on the other hand products were identified as *“healthier products”* when they did comply with the specific nutrient criteria. This was done for each nutrient profile model.

**Table 1:** Comparison of the nutrient profile models of Mexico, WHO Europe and United Kingdom

	Mexican^a^	WHO Europe†	UKNPM‡
*Based on*	Food categories	Food categories	Food or Beverages
*Categories*	12 categories	17 categories	–
*Subcategories*	Subcategories	–	–
*Criteria*	Cut points for nutrition criteria and specified food groups that are completely in or out for food marketing.	Cut points for nutrition criteria and specified food groups that are completely in or out for food marketing.	Score by points • “less healthy” (>4 points for foods and >1 point for beverages)
*Nutrients covered by the model*	Energy, sodium, saturated fat and total sugar	Energy, salt, artificial sweeteners, total fat, saturated fat, total sugar and added sugar	A points • Energy, saturated fat, total sugar and sodium C points • Fruit, vegetables and nuts; fiber and protein.
*Measure*	Serving size	100 g /ml	100 g /ml
*Developed by*	COFEPRIS based on the European pledge	World Health Organization members	Academic researchers by Oxford University

^a^Mexican Ministry of Health. Nutritional criteria for food and non-Alcoholic beverages advertising in television and cinemas targeted to children. Mexico; 2014 [24]. †WHO Regional Office for Europe [28]. ‡ Rayner M, Scarborough P, Boxer A, Stockley L. Nutrient profiles: Development of Final Model. British Heart Foundation Health Promotion Research Group, Department of Public Health, University of Oxford; 2005 [29]

The products advertised were grouped into food categories, using the WHO European model as a reference (Additional file 2). In advertisements with more than one product of the same category, the nutritional content was averaged, and when there were products from different categories, the analysis was made for each product.

### Statistical analysis

The nutritional quality of foods advertised was compared between season (school or vacation), days (weekday or weekend), audiences (general population or 12 years and older) and types of programs (sports, soap opera, cartoons, entertainment, movies and others). A Mann-Whitney *U* test was used to assess differences in nutritional content and quality of the foods advertised between school and vacation season, week and weekend days and different audiences. Differences by type of program were examined through the Kruskal-Wallis test, applying a Bonferroni correction. All results are shown in medians, and the statistical significance was established for a two-tailed test at *p* < 0.05. The analyses were performed using the STATA statistical software package version 12.0 [39].

## Results

During the 600 h of TV recorded, 2,546 food and non-alcoholic beverage advertisements were broadcast, representing (20.7 %) of the total advertisements shown. The advertisements featured 275 different food and beverage products, with a mean frequency of 9.2 advertisements per product and an average of 48.96 food and beverage advertisements per day per channel.

During the school season there were 291.3 advertisements of food and beverages per day, 2.6 times more advertisements as compared with the vacation season where 112.7 advertisements per day were observed (Table 2). Considering that there are five weekdays and that each weekend has only two days, there were 2.4 times more advertisements during weekend days than on weekdays; on average there were 258 food and beverages advertisements per day during weekdays compared with 627 on weekend days.

**Table 2:** Percentage of advertisements for foods and beverages on Mexican TV, by season, day, audience, type of program and nutritional quality

Variables	Number*	Percent
*Season* †		
School	1754	68.9
Average of advertisements per day	291.3	
Vacation	792	31.1
Average of advertisements per day	112.7	
*Day*		
Weekday	1292	50.7
Average of advertisements per day	258.4	
Weekend	1254	49.2
Average of advertisements per day	627	
*Audience* ‡		
General	2217	87.0
12 years and older	329	12.9
*Type of program*		
Sports	140	5.5
Soap opera	514	20.1
Cartoons	348	13.6
Entertainment	525	20.6
Movies	725	28.4
Other	294	11.5
*Nutritional quality*		
* Mexican criteria§*		
Healthier products	910	35.7
Less healthy products	1639	64.3
*WHO Europe ¶*		
Healthier products	429	16.8
Less healthy products	2117	83.1
UKNPM **		
Healthier products	540	21.2
Less healthy products	2006	78.7
* Total*	*2,546*	*100.0*

*n = 2,546 advertisements. † Official calendar Secretary of public Education of Mexico [36]. ‡Federal regulation law on radio and television [37]. §Guidelines, Ministry of Health of Mexico [24]. ¶WHO Regional Office for Europe nutrient profile model [28]. ** United Kingdom Nutrient Profile Model [29]

### Nutritional content of foods and beverages advertised

The median energy and nutrient content of the foods advertised is shown in Table 3. A food and/or beverage advertised on Mexican TV contained a median of 310 kcal of energy, 12 g of sugar, 1 g of fiber, 4 g of total fat, 2 g of saturated fat and 164 mg of sodium per 100 g/ml of product.

**Table 3:** Nutritional content of food and beverage advertisements on Mexican TV, by season, day, audience and type of program

Nutritional content_*_	Total	Season‡			Day‡			Audience‡			Type of program‡						
		School n_**_ 1754	Vacation n_**_ 792	_§_ *p* value	Weekday n_**_1292	Weekend n_**_ 1254	_§_ *p value*	General n_**_2217	12 years and older n_**_329	_§_ *p* value	Sports n_**_140	Soap opera n_**_514	Cartoons n_**_ 348	Entertainment n_**_ 525	Movies n_**_ 725	Others n_**_294	_¶_ *p value*
		M†	M†		M†	M†		M†	M†		M†	M†	M†	M†	M†	M†	
Energy (kcal)	310.0	*318.0*	252.5	<0.001	247.2	*347.5*	<0.001	*310.0*	263.6	0.02	270.0 ^b,c,d,e,f^	224.0 ^a,c,d,e,f^	*367.0* ^a,b,d,e,f^	273.0 ^a,b,c,e,f^	331.0 ^a,b,c,d,f^	305.5 ^a,b,c,d,e^	<0.001
Sugar (g)	12.0	*12.4*	10.0	<0.001	12.0	12.0	0.09	*12.3*	10.0	<0.001	4.5 ^b,c,d,e,f^	10.0 ^a,c,d,e,f^	*30.0* ^a,b,d,e,f^	11.0 ^a,b,c,e,f^	11.0 ^a,b,c,d,f^	12.0 ^a,b,c,d,e^	<0.001
Fiber (g)	1.0	1.0	1.0	0.07	*0.0*	2.0	<0.001	1.0	0.0	0.07	1.7 ^b,c,d,e,f^	*0.0* ^a,c,d,e^	1.5 ^a,b,d,e,f^	1.3 ^a,b,c,e,f^	1.8 ^a,b,c,d,f^	*0.0* ^a,c,d,e,^	<0.001
Total fat (g)	4.0	*6.0*	2.0	<0.001	3.0	*8.2*	<0.001	4.0	6.0	0.57	*9.5* ^b,c,d,f^	2.6 ^a,c,d,e,f^	3.0 ^a,b,d,e,f^	3.0 ^a,b,c,e^	9.0 ^a,b,c,d,f^	4.0 ^a,b,c,e^	<0.001
Saturated fat (g)	2.0	*2.0*	1.0	<0.001	1.0	*4.0*	<0.001	2.0	2.0	0.17	2.2 ^b,c,d,e,f^	1.0 ^a,c,d,e,f^	2.0 ^a,b,e^	1.0 ^a,b,e^	*5.0* ^b,c,d,f^	2.0 ^a,b,e^	<0.001
Sodium (mg)	164.0	*240.8*	96.0	<0.001	100.0	*244.0*	<0.001	*178.0*	132.0	0.01	*412* ^b,c,d,e,f^	100.0 ^a,c,d,e,f^	125.0 ^a,b,e^	125.0 ^a,b,e^	240.8 ^a,b,c,d,f^	163.0 ^a,b,e^	<0.001

*Values measured by 100 g per product*.* † M: median values; ‡total n: 2,546 advertisements; ^a^ Sports ^b^ Soap opera ^c^ Cartoons ^d^ Entertainment ^e^ Movies ^f^ Other; §Mann-Whitney *U* test. ¶Multiple tests with Kruskal-Wallis adjusted with Bonferroni-type; **n: number of advertisements

Significant differences were observed in the median energy, sugar, total fat, saturated fat and sodium content of food and beverages advertised by season, day, audience and type of program (See Table 3). Foods and beverages advertised during school season contained significantly higher amounts of energy, sugar, total fat, saturated fat, and sodium compared to those advertised during the vacation season. Similar results were observed for weekend days compared to weekdays with the exception of sugar, where there were no statistically significant differences. Products advertised during programs targeting a general audience contained more energy, sugar and sodium compared to the programs targeted at an audience 12 years and older. Food products advertised during cartoon programs had the highest energy and sugar content, while those advertised during sports programs had the highest fat and sodium content compared to the other type of programs.

### Nutritional quality of foods and beverages advertised

More than 60.0 % of the foods and beverages advertised on Mexican TV did not meet any of the three nutrient profile model quality standards, as shown in Table 2. 64.3 % of the products advertised did not comply with the Mexican nutritional standards, as compared with 83.1 % and 78.7 % with WHO Europe and UKNPM standards, respectively.

**Fig. 1:**
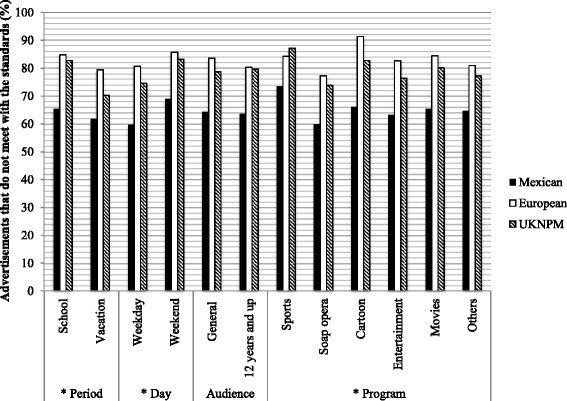
Nutritional quality of food and beverage advertisements on Mexican TV, according to three nutrient profile standards

Figure 1 demonstrates the differences found in the evaluation of the nutritional quality of food and beverages advertised on Mexican TV by season, day and type of program. The percentage of foods advertised that depicted healthier products that met the Mexican, WHO Europe and UKNPM standards was higher in vacation than school season, (38.2 % vs. 34.6 % *p =* 0.07, 20.5 % vs. 15.1 % *p = <0.01* and 29.8 % vs. 17.2 % *p = <0.01,* respectively), and higher during weekdays than on weekend days: (40.3 % vs. 31.0 % *p = <0.01,* 19.2 % vs. 14.2 % *p = <0.01,* and 25.4 % vs. 16.8 % *p = <0.01,* respectively).

During cartoon programming, only 33.9 %, 8.6 %, and 17.2 % of the foods advertised complied with the Mexican, WHO Europe and UKNPM standards. Foods advertised in these types of programs had a lower nutritional quality in the three different criteria, as compared with other types of programming (*p =* <0.05).

### Food and beverage categories most frequently advertised

Considering that some of the advertisements depicted more than one product, there were 17 different food categories, which appeared 2,785 times in 2,546-recorded advertisements. The majority of advertisements were for beverages (24.6 %), followed by chocolate and confectionery (19.7 %), sweet biscuits and pastries (12.0 %), savory snacks (9.3 %), breakfast cereals (7.1 %), ready-made food (6.4 %) and dairy products (6.0 %). The food categories with the lowest percentage of advertisements were: fruit, vegetables and legumes, and meat, poultry and fish. None of the food and beverage TV advertisements were for fresh fruit, vegetables, or legumes. Additionally, the results demonstrate that Mexican standards allow a higher proportion of cereals (75.8 %), yogurts (52.7 %), and cakes, biscuits and pastries (38.4 %) to be advertised, compared to European and UKNP models. The results are shown in Table 4.

**Table 4:** Percentage of food and beverage advertisements on Mexican TV, that do not comply with the nutrient profile standards, by food category

Categories	Total		*"Less healthy"*					
			*Mexican standards*		WHO* Europe standards		UKNPM* standards	
	*n* ^†^	%	*n* ^†^	%	*n* ^†^	%	*n* ^†^	%
Beverages	686	24.6	439	63.9	514	74.9	447	65.1
Chocolate and sugar confectionery	549	19.7	493	89.8	549	100	464	84.5
Cakes, biscuits and pastries	336	12	207	61.6	336	100	336	100
Savory snacks	261	9.3	247	94.6	261	100	261	100
Breakfast cereal	198	7.1	48	24.2	197	99.4	198	100
Ready-made food and dishes	179	6.4	125	69.8	125	69.8	100	55.8
Yoghurts, milk and similar foods (dairy)	167	6	79	47.3	156	93.4	165	98.8
Sauces, dips and dressings	89	3.2	80	89.8	68	76.4	49	55
Bread, bread products and crisp breads	85	3	52	61.1	23	27	68	80
Fresh or dried pasta, rice and grains	82	2.9	12	14.6	0	0	28	34.1
Edible ices	57	2	22	38.6	57	100	40	70.1
Butter and other fats and oils	40	1.4	15	37.5	15	37.5	40	100
Cheese	30	1	30	100	29	96.6	30	100
Processed meat, poultry, fish and similar	21	0.7	9	42.8	9	42.8	16	76.1
Processed fruit, vegetables and legumes	3	0.1	3	100	3	100	3	100
Meat, poultry, fish and similar	2	0	0	0	0	0	0	0
Fresh and frozen fruit, vegetables and legumes	0	0	0	0	0	0	0	0
*Total*	*2,785*	100	*1861*	*66.8*	*2342*	*84*	*2245*	*80.6*

*WHO “World Health Organization”; UKNPM “United Kingdom Nutrient Profile Model”; Categories according to the WHO Regional Office for Europe nutrient profile model [28], shown in supplementary material 2.†n: number of advertisements

**Fig. 2:**
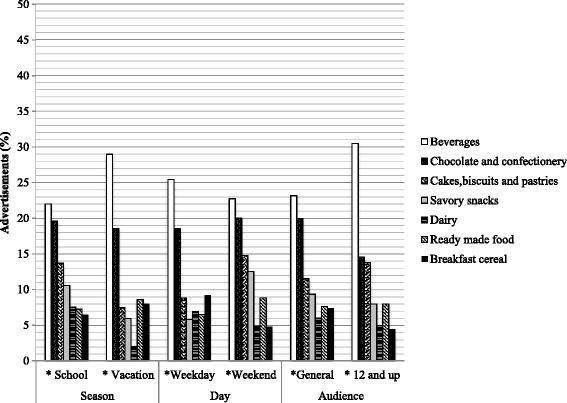
Comparison of the most shown categories advertisements on Mexican TV, by season, day and audience

**Fig. 3:**
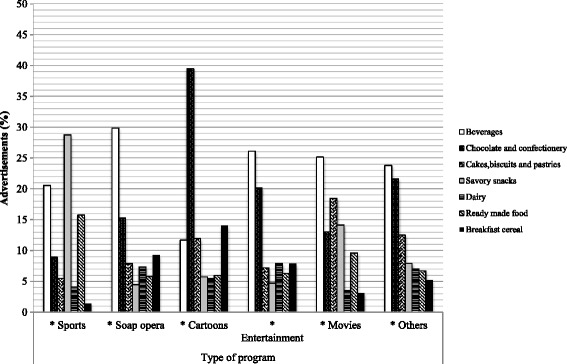
Comparison of the most shown categories of food and beverage advertisements on Mexican TV, by type of program

Categories of products advertised the most, did not differ significantly by season, day or audience, as shown in Fig. 2. Beverages were the most popular category, although they were shown significantly more often during vacation season (28.9 %, *n* = 249) compared to school season (21.9 %, *n =* 437); on weekdays (25.4 %, *n* = 361) compared to weekend days (22.7 %, *n* = 325); and for an audience of 12 years and up (30.4 %, *n* = 111) compared to a general audience (23.1 %, *n* = 575). Based on type of program shown in Fig. 3, only sports programming had the majority of advertisements for the savory snacks category (28.7 %, *n* = 42). In cartoons the majority of advertisements were for the chocolate and confectionery category (39.4 %, *n* = 152). For other types of programming (soap opera, entertainment, movies and others) beverages remained the most popular.

Table 4 shows the percentage of “less healthy” products depicted in advertisements, namely those that do not comply with the nutrient profile standards of Mexico, WHO Europe and UKNPM by food category. Apart from fresh produce, the percentage of foods advertised that complied with the standards was higher within the food category of fresh or dried pasta, rice and grain products. Within this category, 85.3 % of products complied with the Mexican standards, 100 % with the European, and 65.8 % with the UKNPM. In addition, the three different standards considered all the meat, poultry, fish and similar products shown in Mexican TV as healthier products.

## Discussion

This study is the first one to examine the nutritional quality of food and beverages advertised on Mexican TV, analyzed according to the Mexican, WHO Europe and UK nutrient profile models. More than 60 % of the foods and beverages advertised on Mexican TV did not comply with any of the nutritional quality standards, and thus should not have been marketed to children. According to the three models, the food products advertised on Mexican TV had a poor nutrient profile, especially during cartoons and sports programming.

Our findings are similar to what previous studies have found in other countries. Diverse reports have found that the nutritional content of products advertised on TV is more frequently high in HFSS and most of them are targeted during children’s programming [18, 40, 41]. These findings are also demonstrated in the current study; advertisements with the highest amount of sugar and energy were broadcast in cartoons, program classified appropriate for children. This can be explained by the fact that 39.4 % of the advertisements shown during cartoons fell into the chocolate and sugar confectionery category. Our results for fat and sodium differed from other studies as we showed that the advertisements for products with the highest amount of fat and sodium were shown during sports programming. This can be explained by the fact that 28.7 % of the advertisements shown during this type of programming were for snack foods.

On average, beverages, candy, snacks, breakfast cereals, dairy, and prepared foods (including fast food) accounted for the most advertisements shown on TV. International studies also suggest that these categories are the most frequently advertised on TV [16, 30, 41, 42]. It is important to note that the products in these categories often have considerable amounts of energy, sugar, fat and sodium [34, 42]. In contrast, in the present study, the most nutritious categories (fresh and frozen fruit, vegetables and legumes, meat, poultry, fish and similar) represented less than 1 % of advertisements, and were significantly less likely to appear in advertisements compared to other categories.

Findings about food and beverage advertisement shown more often during school season could not be explained with scientific evidence. Nevertheless, during this period, people are in a routine and allocate specific time to watch TV; we can assume that food marketing is higher compared to those days where people abandon their routine, such as vacation season.

Results of the evaluation of the nutritional quality based on UKNPM were consistent with other recent study results. In Spain in 2012, 61.5 % of the products advertised were less healthy [34], while in New Zealand in 2007 the proportion was 66.3 % [43]. In Canada and the UK in 2006, the proportion of less healthy food advertised on TV was 65.7 % and 54.5 %, respectively [44]. In the present study, it was observed that in Mexico 78.79 % of the advertisements contained products that cannot be advertised according to this standard. Brinsden and Lobstein in 2013 reported that in the United States 63 % of the products advertised were less healthy products [45]. The researchers also compared different nutrient profile schemes, and found that, though the proportion of products that were allowed to be advertised varies according to each standard, it was still the minority of advertised products that were of high enough nutrition quality to be advertised. These results were similar to the current study.

Studies have found links between TV food advertising and consumption. They consistently demonstrate that food and beverage advertisements on TV influence children’s food preferences, purchasing requests and eating behaviors, which has harmful effects on young people’s diets, and can cause obesity and related diseases [5, 13, 46, 47]. Further evidence demonstrates that it is extraordinarily difficult to counteract the influence of food marketing, even for adults, especially when it promotes HFSS products [48].

There is little documentation on the effect of regulation on TV advertising [49, 50], but modeling projections indicate that regulation would be a cost-effective strategy [51, 52]. Mexico has introduced a regulation regarding TV advertising of food and beverages to children. This study shows that the foods and beverages advertised on Mexican TV do not meet the regulations. Moreover, the poor nutritional quality of the majority of food and beverage products in advertisements on TV has left Mexican children exposed to substantial amounts of unhealthy food advertising, which likely contributes to obesity. While these regulations are a good first step, Mexico needs stricter regulations with nutrient profile standards similar to WHO and UK. The Mexican standards have the least strict nutrition criteria among the three, leaving products with a considerably high amount of energy, sugar and fat being advertised to children; especially in food categories, such as cereals, yogurts and cakes. These results suggest that, if the Mexican criteria reinforce the cut off points for sugar, it would improve the quality of products advertised on television. Additionally, this strategy needs to be supported by complementary actions such as, reformulation of foods and beverages, prepackaging portion sizes, raising awareness and education.

Of course, given the multiple channels of marketing such as school marketing, movies, social media, and supermarkets; TV is just one of the many sources of food and beverage advertisements that can influence children’s purchase behavior. However, TV viewing has been firmly linked to childhood obesity, through the effect of the power of advertisements for HFSS foods on TV. Furthermore, there is evidence that documents little progress in reducing unhealthy food advertising to children under age 12 [53, 54].

This is the first study that provides information on the nutritional content of Mexican TV with national data before the regulation started in 2015. The results can be useful in evaluating the changes in food and beverage advertisements in Mexican TV before and after the regulation started.

Future work could investigate the exposure of the Mexican population to the food and beverages advertised, and the power of the advertisements (promotional characters, premium offers) and evaluate the effect that they may have had on Mexican population. Additionally, further studies should also address whether the Mexican regulation is working and examine the difference between Mexican children’s actual exposure to unhealthy food advertising under the current law and what it would have been with other standards (WHO or UK) during the same time.

Our findings are subject to limitations. First, the present study does not evaluate product placements within the TV shows, although this data covered the majority of the products advertised on Mexican TV. Channels were selected based on general data instead of audience data specifically for children, so there is no data on children’s peak and non peak viewing times to compare. Furthermore, the nutritional composition data for products was collected more recently than the advertisement recordings, which may have created small discrepancies among the data collected from nutritional content presented in advertisements. The strengths of the present study are its large sample sizes, 600 h recorded from channels with very high rating at national level. Thus, it is unlikely that a different selection of TV channels would change the findings, because both the number and type of advertisements are similar on the different channels. Another strength of this research is its objective approach, which uses different quality standards, and provides evidence and results that are therefore comparable with others studies from foreign countries.

## Conclusions

In summary, this study is the first one to examine the nutritional quality of foods and beverage products advertised on Mexican TV. We conclude that the majority of food and beverage advertisements aired do not comply with any nutrition quality standard and were considered to promote less healthy products, especially during cartoons and sports programming. We suggest a need for constructive engagement with the government to improve and strengthen the regulation of food and beverages aimed at children. Progress in policy development may be difficult, specifically classifying products for which marketing should be restricted. Although nutrient profile models could help, there are international recommendations that consider it unsuitable to advertise any kind of industrialized foods to children [22]. However, the use of the UKNPM or European standards to reinforce the Mexican nutritional standard, the one with the most lax criteria should be considered, especially to improve sugar cut off points. TV is one of the many screen-based advertising vehicles that contribute to children’s exposure to HFSS food and beverages. Thus, regulatory strategies on the topic should be supported by other public health actions to promote healthy environments.

## Abbreviations

ENSANUT, National Health and Nutrition Survey; HFSS, high in saturated fat, sugar and/or sodium; IOTF, International Obesity Taskforce; NCD, Non-communicable disease; PHAO, Pan American Health Organization; TV, television; UKNPM, United Kingdom Profile Model; WHO, World Health Organization

## Additional files

Additional file 1: Mexican nutrition criteria for food and beverage advertised on TV and cinemas [24]. (DOCX 23 kb) Additional file 2: WHO Europe Nutrient Profile Model [28]. (DOCX 19 kb)
