# Supplementary material for: Nutritional quality of foods and non-alcoholic beverages advertised on Mexican television according to three nutrient profile models
Source: BMC Public Health. 2016 Aug 5;16:733. doi: 10.1186/s12889-016-3298-0 (PMC4975920; doi:10.1186/s12889-016-3298-0)
Supplement: Additional file 1: — Mexican nutrition criteria for food and beverage advertised on TV and cinemas [24]. (DOCX 23 kb) [file 12889_2016_3298_MOESM1_ESM.docx]

**Additional file 1.** Mexican nutrition criteria for food and beverage advertised on TV and cinemas[24].

| **Categories** | | | | |
| --- | --- | --- | --- | --- |
| **Categories and subcategories** | **Energy** | **Sodium** | **Saturated Fat** | **Total sugar** |
| Category 1. | Oils and fat | | | |
| Subcategory A. | Vegetable oils and butters | | | |
|  | Energy (kcal/portion) | Sodium mg/ on 100g or 100 ml | Saturated fat g/ on 100g or 100 ml | Sugar g/ on 100g or 100ml |
| 10g | 85 | 500 | 33% of total fat | 5 |
| Subcategory B | Emulsions (mayonnaise, mayonnaise dressings and salad dressings). | | | |
|  | Energy (kcal/portion) | Sodium mg/ on 100g or 100 ml | Saturated fat g/ on 100g or 100 ml | Sugar g/ on 100g or 100ml |
| 15g | 85 | 750 | 33% of total fat | 5 |
| Category 2. | Vegetables, fruits, legumes, nuts, seeds and tubers (except the processed ones for snacks) | | | |
| Subcategory A | Vegetables, fruits, legumes, tubers, soy solid foods; frozen, canned and dehydrated | | | |
|  | Energy (kcal/portion) | Sodium mg/ on 100g or 100 ml | Saturated fat g/ on 100g or 100 ml | Sugar g/ on 100g or 100ml |
| 110g | 170 | 300 | 1.5 | 15 |
| Subcategory B | Soy liquid foods with or with out juice | | | |
|  | Energy (kcal/portion) | Sodium mg/ on 100g or 100 ml | Saturated fat g/ on 100g or 100 ml | Sugar g/ on 100g or 100ml |
| 200 ml | 140 | 110 | 0.5 | 9 |
| Subcategory C | Juice | | | |
|  | Energy (kcal/portion) | Sodium mg/ on 100g or 100 ml | Saturated fat g/ on 100g or 100 ml | Sugar g/ on 100g or 100ml |
| 250 ml | 130 | 10 | N/A | 13 |
| Subcategory D | Nectar juice | | | |
|  | Energy (kcal/portion) | Sodium mg/ on 100g or 100 ml | Saturated fat g/ on 100g or 100 ml | Sugar g/ on 100g or 100ml |
| 200ml | 104 | 28 | N/A | 13 |
| Subcategory E | Sauces based on fruits/vegetables/legumes | | | |
|  | Energy (kcal/portion) | Sodium mg/ on 100g or 100 ml | Saturated fat g/ on 100g or 100 ml | Sugar g/ on 100g or 100ml |
| 100 | 100 | 500 | 1.5 | 10 |
| Subcategory F | Spices based on fruits/vegetables/legumes | | | |
|  | Energy (kcal/portion) | Sodium mg/ on 100g or 100 ml | Saturated fat g/ on 100g or 100 ml | Sugar g/ on 100g or 100ml |
| 20g | 85 | 750 | 1.5 | 10 |
| Subcategory G | Nuts and seeds | | | |
|  | Energy (kcal/portion) | Sodium mg/ on 100g or 100 ml | Saturated fat g/ on 100g or 100 ml | Sugar g/ on 100g or 100ml |
| 30g | 200 | 670 | 10 | 15 |
| Subcategory H | Spreadable of nut and seed | | | |
|  | Energy (kcal/portion) | Sodium mg/ on 100g or 100 ml | Saturated fat g/ on 100g or 100 ml | Sugar g/ on 100g or 100ml |
| 30g | 200 | 670 | 10 | 15 |
| Category 3. | Meat products | | | |
|  | Energy (kcal/portion) | Sodium mg/ on 100g or 100 ml | Saturated fat g/ on 100g or 100 ml | Sugar g/ on 100g or 100ml |
| 45 g | 170 | 800 | 6 | 5 |
| Category 4. | Fish products | | | |
| Subcategory A | Fish and seafood products | | | |
|  | Energy (kcal/portion) | Sodium mg/ on 100g or 100 ml | Saturated fat g/ on 100g or 100 ml | Sugar g/ on 100g or 100ml |
| 50 g fish  100 g seafood | 170 or > 170 if 25% of total fat are from polyunsaturated fat | 450 | 33% of total fat, including trans fat | 5 |
| Category 5. | Dairy | | | |
| Subcategory A | Milk products, yoghurts, fermented product, petit Suisse, milk candy, milk base jello, powder milk a base de | | | |
|  | Energy (kcal/portion) | Sodium mg/ on 100g or 100 ml | Saturated fat g/ on 100g or 100 ml | Sugar g/ on 100g or 100ml |
| 240 ml for liquid products  200 ml/g for yoghurts to drink  100g for solids | 170 | 300 | 2.6 | 13.5 |
| Subcategory B | Fresh cheese (panela, Oaxaca, etc..) | | | |
|  | Energy (kcal/portion) | Sodium mg/ on 100g or 100 ml | Saturated fat g/ on 100g or 100 ml | Sugar g/ on 100g or 100ml |
| 30g | 70 | 800 | 10 | 8 |
| Subcategory C | Hard cheese | | | |
|  | Energy (kcal/portion) | Sodium mg/ on 100g or 100 ml | Saturated fat g/ on 100g or 100 ml | Sugar g/ on 100g or 100ml |
| 30g | 85 | 900 | 15 | 5 |
| Subcategory D | Processed and cream cheese | | | |
|  | Energy (kcal/portion) | Sodium mg/ on 100g or 100 ml | Saturated fat g/ on 100g or 100 ml | Sugar g/ on 100g or 100ml |
| 30g | 170 | 800 | 10 | 8 |
| Category 6. | Cereals and tuber products | | | |
| Subcategory A | Cakes | | | |
|  | Energy (kcal/portion) | Sodium mg/ on 100g or 100 ml | Saturated fat g/ on 100g or 100 ml | Sugar g/ on 100g or 100ml |
| 45g | 190 | 450 | 10 | 30 |
| Subcategory B | Sweet bread | | | |
|  | Energy (kcal/portion) | Sodium mg/ on 100g or 100 ml | Saturated fat g/ on 100g or 100 ml | Sugar g/ on 100g or 100ml |
| 50g | 190 | 450 | 10 | 30 |
| Subcategory C | Sweet cookies | | | |
|  | Energy (kcal/portion) | Sodium mg/ on 100g or 100 ml | Saturated fat g/ on 100g or 100 ml | Sugar g/ on 100g or 100ml |
| 30g | 160 | 450 | 10 | 30 |
| Subcategory D | Cereal bars | | | |
|  | Energy (kcal/portion) | Sodium mg/ on 100g or 100 ml | Saturated fat g/ on 100g or 100 ml | Sugar g/ on 100g or 100ml |
| 30g | 160 | 450 | 10 | 35 |
| Subcategory E | Breakfast cereal | | | |
|  | Energy (kcal/portion) | Sodium mg/ on 100g or 100 ml | Saturated fat g/ on 100g or 100 ml | Sugar g/ on 100g or 100ml |
| 30g | 210 | 500 | 5 | 30 |
| Subcategory F | Rice, pasta, bread, crackers | | | |
|  | Energy (kcal/portion) | Sodium mg/ on 100g or 100 ml | Saturated fat g/ on 100g or 100 ml | Sugar g/ on 100g or 100ml |
| 50g | 340 | 500 | 5 | 5 |
| Subcategory G | Corn and flour tortillas; flat breads | | | |
|  | Energy (kcal/portion) | Sodium mg/ on 100g or 100 ml | Saturated fat g/ on 100g or 100 ml | Sugar g/ on 100g or 100ml |
| 50g | 300 | 670 | 5 | 4 |
| Category 7. | Soups, composed dishes, filled sandwiches, spices and condiments | | | |
| Subcategory A | Soups, spices and condiments | | | |
|  | Energy (kcal/portion) | Sodium mg/ on 100g or 100 ml | Saturated fat g/ on 100g or 100 ml | Sugar g/ on 100g or 100ml |
| 200ml | 170 | 350 | 1.5 | 7.5 |
| Subcategory B | Composed dishes, filled sandwiches and main course dishes | | | |
|  | Energy (kcal/portion) | Sodium mg/ on 100g or 100 ml | Saturated fat g/ on 100g or 100 ml | Sugar g/ on 100g or 100ml |
| 200g | 425 | 400 | 5 | 7.5 |
| Category 8. | Dessert | | | |
| Subcategory A | Edible ices | | | |
|  | Energy (kcal/portion) | Sodium mg/ on 100g or 100 ml | Saturated fat g/ on 100g or 100 ml | Sugar g/ on 100g or 100ml |
| 76g/75 ml | 110 | 120 | 5 | 20 |
| Subcategory B | Jello | | | |
|  | Energy (kcal/portion) | Sodium mg/ on 100g or 100 ml | Saturated fat g/ on 100g or 100 ml | Sugar g/ on 100g or 100ml |
| 130g | 110 | 120 | 5 | 20 |
| Category 9. | Flavored beverages | | | |
| Subcategory A | Flavored beverages with low energy content | | | |
|  | Energy (kcal/portion) | Sodium mg/ on 100g or 100 ml | Saturated fat g/ on 100g or 100 ml | Sugar g/ on 100g or 100ml |
| 200ml | 40 | 28 | N/A | 5 |
| Category 10. | Snacks | | | |
|  | Energy (kcal/portion) | Sodium mg/ on 100g or 100 ml | Saturated fat g/ on 100g or 100 ml | Sugar g/ on 100g or 100ml |
| 30g | 170 | 670 | 6.3 | 10 |
| Category 11. | Confectionery products | | | |
|  | Energy (kcal/portion) | Sodium mg/ on 100g or 100 ml | Saturated fat g/ on 100g or 100 ml | Sugar g/ on 100g or 100ml |
| 15g | N/A | N/A | N/A | N/A |
| Category 12. | Chocolate and similar products | | | |
|  | Energy (kcal/portion) | Sodium mg/ on 100g or 100 ml | Saturated fat g/ on 100g or 100 ml | Sugar g/ on 100g or 100ml |
| 30g | N/A | N/A | N/A | N/A |

* Items not specifically framed in any category referred took the most similar. † It is allowed to advertise at any time water, food for toddlers and babies, chewing gum and breath mints without sugar.

‡ It is allowed to advertise confectionery and chocolate products, and all other products that not comply with the nutrition criteria during: weekdays 12am to 2:30 pm and 7:30pm to 11:59pm; weekend days 12am to 7pm and 7:30pm to 11:59pm; or during programs targeted towards 12 years and older audience. § The analysis for the present study was done for 100g per product.
