# Supplementary material for: Nutritional quality of foods and non-alcoholic beverages advertised on Mexican television according to three nutrient profile models
Source: BMC Public Health. 2016 Aug 5;16:733. doi: 10.1186/s12889-016-3298-0 (PMC4975920; doi:10.1186/s12889-016-3298-0)
Supplement: Additional file 2: — WHO Europe Nutrient Profile Model [28]. (DOCX 19 kb) [file 12889_2016_3298_MOESM2_ESM.docx]

**Additional file 2.** WHO Europe Nutrient Profile Model [28].

| **Food category** | **Included in category (examples)** | **Not included in category (examples)** | **Marketing not permitted if product exceeds, per 100 g:** | | | | | | | | | | | | |
| --- | --- | --- | --- | --- | --- | --- | --- | --- | --- | --- | --- | --- | --- | --- | --- |
|  |  |  | **Total fat (g)** | **Saturated fat (g)** | | | **Total sugars (g)** | | **Added sugars (g)** | | **Non-sugar sweeteners (g)** | ^*^**Salt**  **(g)** | **Energy (kcal)** | | |
| 1. Chocolate and sugar confectionery, energy bars, and sweet toppings and desserts (Chocolate and confectionery) | Chocolate and other products containing cocoa; white chocolate; jelly, sweets, chewing gum; caramels; spreadable chocolate and other sweet toppings. | Chocolate flavored breakfast cereals; cakes and pastries; biscuits and other covered in chocolate | ^†^ Not permitted | | | | | | | | | | | | |
| 2. Cakes, sweet biscuits and pastries; other sweet bakery wares, and dry mixes for making such (Cakes,biscuits and pastries) | Pastries; croissants; cookies/ biscuits; sponge cakes; wafers; fruit pies; sweet buns; chocolate-covered biscuits; cake mixes and batters | Bread and bread products | ^†^ Not permitted | | | | | | | | | | | | |
| 3.Savory snacks | Popcorn; seeds; nuts and mixed nuts; savory biscuits and pretzels; other snacks made from rice, maize, dough or potato |  |  | |  | | |  | | 0 |  | 0.1 |  | |  |
| 4. Beverages | | | | | | | | | | | | | | | |
| a) Juices | 100% fruit and vegetable juices; juices reconstituted from concentrate and smoothies |  | ^†^ Not permitted | | | | | | | | | | | | |
| b) Milk drinks | Milks and sweetened milks; almond, soya, rice and oat milks | Cream | 2.5 |  | | | |  | | 0 | 0 |  |  | | |
| c) Energy drinks |  |  | ^†^ Not permitted | | | | | | | | | | | | |
| d)Other beverages | Cola, lemonade, orangeade; other soft drinks, sweetened beverages, mineral and/or flavored waters with added sugars or sweetener | 100% fruit and vegetable juices; milk drinks |  |  | | | |  | | 0 | 0 |  | |  | |
| 5. Edible ices | Ice cream frozen yoghurt, iced lollies and sorbets |  | ^†^ Not permitted | | | | | | | | | | | | |
| 6.Breakefast cereals | Oatmeal; cornflakes; chocolate breakfast cereals; mueslis |  | 10 | | |  | | 15 | |  |  | 1.6 | |  | |
| 7. Yoghurts, sour milk, cream and other similar foods (Dairy) | Yoghurt; kephir; buttermilk; flavored sour, fermented milk and drinking yoghurt; cheese-based and other yoghurt substitutes; yoghurt products containing additional ingredients (such as fruit; muesli); cream. | Milks and sweetened milks; almond, rice and oat milks | 2.5 | | | 2.0 | | 10 | |  |  | 0.2 | |  | |
| 8. Cheese | Medium-hard and hard cheeses; soft cheeses; fresh cheese (such as ricotta, mozzarella); grated or powdered cheese; cottage cheese; processed cheese spreads |  | 20 | | |  | |  | |  |  | 1.3 | |  | |
| 9.Ready-made and convenience foods and composite dishes | Pizzas; lasagna and other pasta dishes with sauces; quiches; ready meals; ready-made sandwiches, burgers, pizzas; fast food; filled pastas; soups, rice and stews (packaged or tinned); mixes and dough |  | 10 | | | 4 | | 10 | |  |  | 1 | | 225 | |
| 10. Butter and other fats and oils | Butter; vegetable oils, margarines and spreads |  |  | | | 20 | |  | |  |  | 1.3 | |  | |
| 11. Bread, bread products and crisp breads | Ordinary bread (containing cereal, leavens and salt); gluten-free bread; unleavened bread; crisp breads; rusks and toasted breads | Sweet biscuits; pastries; cakes | 10 | | |  | | 10 | |  |  | 1.2 | |  | |
| 12.Fresh or dried pasta, rice and grains |  | Filled pasta and pasta in sauce | 10 | | |  | | 10 | |  |  | 1.2 | |  | |
| 13. Fresh and frozen meat, poultry, fish and similar | Eggs |  | ^†^ Permitted | | | | | | | | | | | | |
| 14. Processed meat, poultry, fish and similar | Sausage, ham, bacon; chicken nuggets; smoked and pickled fish  tinned fish in brine or oils; fish fingers and breaded/battered  fish | Pepperoni pizza | 20 | | |  | |  | |  |  | 1.7 | |  | |
| 15. Fresh and frozen fruit, vegetables and legumes | Fruit and vegetables; legumes; starchy vegetables, roots and tubers | Tinned fruits, vegetables and legumes; fruit in syrup; dried fruit; frozen fruit with added sugar | ^†^ Permitted | | | | | | | | | | | | |
| 16. Processed fruit, vegetables and legumes | Tinned fruit, vegetables and legumes; marmalade; jams; pickled vegetables and fruit; stewed fruits; fruit peel; frozen French fries; frozen fruit with added sugar | Fruit juice | 5 | | |  | | 10 | | 0 |  | 1 | |  | |
| 17. Sauces, dips and dressings | Salad dressings; tomato ketchup; mayonnaise; ready to-use dips; soya sauce; mustard and mustard flour |  | 10 | | |  | |  | | 0 |  | 1 | |  | |

*Salt-1g of sodium is equivalent to about 2.5 g of salt. †According to the model, marketing for five categories is not permitted, and two always permitted, meaning that no nutrient criteria are required. ‡Determining whether a food product may or may not be marketed the product must not exceed the specific criteria; if an advertisement includes more than one product, all items must individually meet the relevant nutrient criteria.
